# Development and initial testing of a computer-based patient decision aid to promote colorectal cancer screening for primary care practice

**DOI:** 10.1186/1472-6947-5-36

**Published:** 2005-11-28

**Authors:** Jane Kim, Annie Whitney, Sarah Hayter, Carmen Lewis, Marci Campbell, Lisa Sutherland, Beth Fowler, Sue Googe, Regina McCoy, Michael Pignone

**Affiliations:** 1 Preventive Medicine Residency Program, University of North Carolina, Chapel Hill, NC, USA; 2 Division of General Internal Medicine, Department of Medicine, University of North Carolina, Chapel Hill, NC, USA; 3 Department of Nutrition, University of North Carolina School of Public Health, Chapel Hill, NC, USA; 4 Lineberger Comprehensive Cancer Center, University of North Carolina, Chapel Hill, NC, USA

## Abstract

**Background:**

Although colorectal cancer screening is recommended by major policy-making organizations, rates of screening remain low. Our aim was to develop a patient-directed, computer-based decision aid about colorectal cancer screening and investigate whether it could increase patient interest in screening.

**Methods:**

We used content from evidence-based literature reviews and our previous decision aid research to develop a prototype. We performed two rounds of usability testing with representative patients to revise the content and format. The final decision aid consisted of an introductory segment, four test-specific segments, and information to allow comparison of the tests across several key parameters. We then conducted a before-after uncontrolled trial of 80 patients 50–75 years old recruited from an academic internal medicine practice.

**Results:**

Mean viewing time was 19 minutes. The decision aid improved patients' intent to ask providers for screening from a mean score of 2.8 (1 = not at all likely to ask, 4 = very likely to ask) before viewing the decision aid to 3.2 afterwards (difference, 0.4; p < 0.0001, paired t-test). Most found the aid useful and reported that it improved their knowledge about screening. Sixty percent said they were ready to be tested, 18% needed more information, and 22% were not ready to be screened. Within 6 months of viewing, 43% of patients had completed screening tests.

**Conclusion:**

We conclude that a computer-based decision aid can increase patient intent to be screened and increase interest in screening. Practice Implications: This decision aid can be viewed by patients prior to provider appointments to increase motivation to be screened and to help them decide about which modality to use for screening. Further work is required to integrate the decision aid with other practice change strategies to raise screening rates to target levels.

## Background

Colorectal cancer (CRC) is the second leading cause of cancer death and third most diagnosed cancer in the United States [1,2]. Multiple policy-making organizations have published evidence-based CRC screening guidelines that recommend annual fecal occult blood testing (FOBT), sigmoidoscopy or barium enema every 5 years, or colonoscopy every 10 years for adults 50 and older [3-5]. Despite these guidelines, rates of colorectal cancer screening in the U.S. are low with only approximately 50% of adults 50 and older reporting a CRC screening test within recommended time intervals [6,7].

Given the many testing options, colorectal cancer screening can be a complex issue, and time limitations may prevent providers from adequately discussing all options with patients. Patient decision aids offer a means of circumventing these limitations. Decision aids are educational materials that help individuals understand their choices for screening or treatment [8,9]. They have been shown to improve patients' knowledge, reduce decisional conflict, and increase active patient participation in medical decision making [8].

We previously conducted a randomized controlled trial of an 11-minute videotape-based decision aid on CRC screening paired with a color-coded chart marker and brochure [10]. The decision aid was based on the transtheoretical stages of change model [11]. Patients indicated their stage of readiness to be screened by selecting a color-coded brochure, and a chart marker of the same color was attached to their charts. The decision aid increased patients' intent to ask their providers for screening, and 47% of intervention group participants had CRC screening tests ordered compared to 26% of controls. A chart review 3 months afterwards found increased screening test completion in the intervention group compared to controls (37% vs. 23%, respectively, p < 0.05).

Despite the videotape-based decision aid's success in raising screening rates, there were some limitations in its format and content. First, it only offered FOBT and sigmoidoscopy; colonoscopy was not an available option at the time of the original study. Because it was produced in videotape form, the decision aid could not be easily updated to include newly endorsed screening modalities or other new information. In addition, the videotape could not be tailored to meet different levels of patient interest in CRC screening. Recent advances in web-based technology made it possible to produce a computer-based decision aid that can be updated and customized to meet patients' individual knowledge needs.

We sought to develop, refine, and pilot test whether a computer-based, patient-directed decision aid can increase patient interest in screening, meet informational needs, and lead to the ordering and completion of colorectal cancer screening tests.

## Methods

### Computer-based decision aid development

We based the content of the computer-based decision aid, called CHOICE™ version 1.0, on systematic reviews of the literature and our videotape decision aid [10]. New segments on colonoscopy, barium enema, and comparative information about the tests were developed. We also developed patient vignettes by filming interviews with patients who had undergone screening and agreed to discuss their experiences. The decision aid, two self-administered questionnaires, and a data collection mechanism using Microsoft Access as a back-end database were programmed into a web-based format using Active Server Pages. The decision aid was developed for use on a local computer version, although an Internet-based version has subsequently been developed.

### Decision aid format and content

The final version of the decision aid consists of a modular design with a 5-minute introduction and five additional 3–5 minute segments that describe individual screening tests or comparative information about the tests. An audio track accompanies the entire decision aid and explains all figures that are presented, making the content accessible to users with varying levels of literacy. Readability was not formally assessed given that the decision aid was not based on prose text. However, we designed the interface, including the audio track and figures, with the intent to make the information easy to understand. The patient uses a mouse to activate an introductory segment that explains what colorectal cancer is, describes its prevalence and lifetime risk, outlines the importance of colorectal cancer screening and the benefits of early detection, and gives a brief overview of each recommended screening modality. A physician provided narration and multiple interviews were included of patients describing their experiences and views of colorectal cancer screening. This portion of the decision aid was to be viewed by all users.

**Figure 1:**
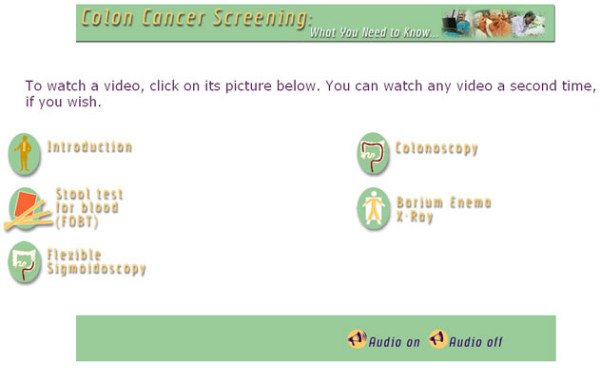
Menu of choices.

**Figure 2:**
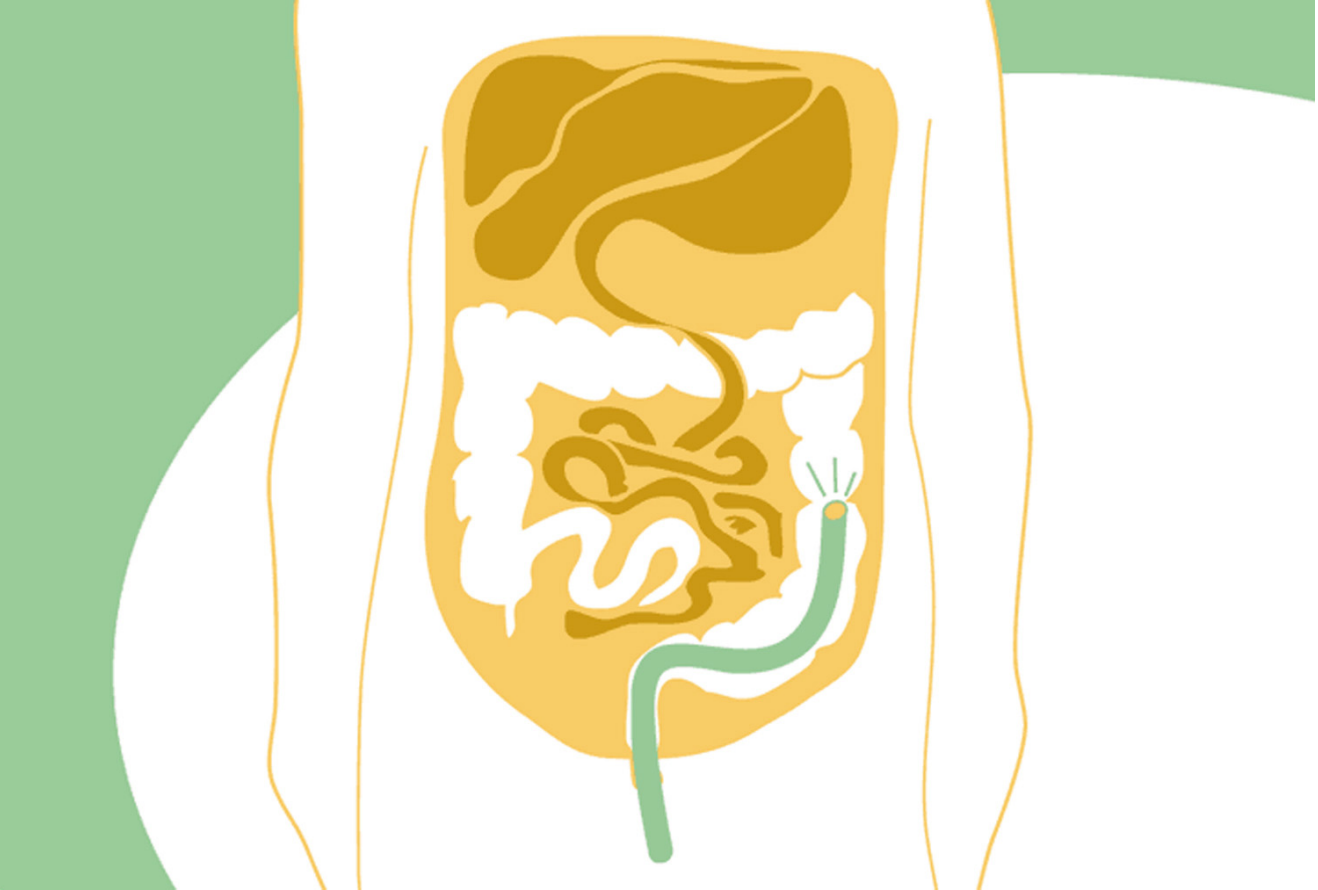
Illustration of endoscopy technique.

**Figure 3:**
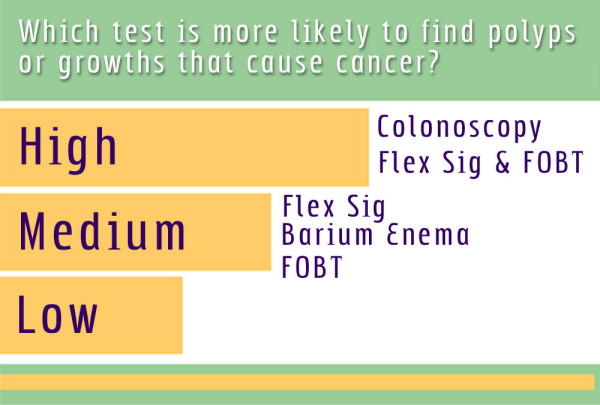
Comparison of screening tests.

After the introduction, the decision aid directs patients to a menu of choices that allows them to choose one or more additional test-specific video segments by clicking on the name of the test with the mouse (Figure 1). Each segment contains footage that utilizes physician and patient narration and describes how each test is performed, the preparation required, and common patient concerns about the procedure. While the physician narrates, animated cartoons (Figure 2) and text visually depict the anatomic area examined by the test and the way the screening test is performed. Our aim was to present a balanced overview of the risks and benefits of each test, including preparation required, discomfort anticipated during the test, the risk of adverse effects, as well as the ability of each test to detect colorectal cancer or precancerous polyps. After the overview about the test, the decision aid presents multiple 5–15 second patient vignettes. These vignettes consist of interviews with white and African American men and women describing their reasons for choosing a particular screening modality, their experiences with the test, and the benefits and downsides of the test. Additional video footage shows patients preparing for the tests. Follow-up of abnormal results and comparisons of different screening modalities to one another are explained via text, narration, and graphs (Figure 3). At the end of each segment, patients are directed to the navigation screen that allows them to choose another test information segment, examine comparative information, or to go to the post-intervention questionnaire. After initial testing, the decision aid was modified to require that patients watch the introduction and at least one other video segment.

### Usability testing

We recruited twelve patients 50–75 years old to participate in each round of usability testing. These patients were a convenience sample recruited from the University of North Carolina-Chapel Hill (UNC) general internal medicine practice. We attempted to identify users with a wide range of previous computer experience and education levels. The computer used for the usability testing and the pilot study was an IBM PC with a Pentium processor and Windows operating system. Research team members experienced in usability testing observed and videotaped patients as they worked with the decision aid using the Think Aloud technique [12]. The study investigators modified the decision aid via an iterative process based on feedback from the usability testing that identified users' difficulties with the decision aid. In the first session, users, particularly those less familiar with computers, had several problems with navigation. These problems included confusion about how to use the mouse, difficulty in reading the type font, and inability to move between screens. Based on these results, we increased font sizes, changed the placement and color contrast of navigation elements and content, and simplified navigation options and operation of the video segments. After these changes, patients in the second round of testing with varying levels of computer experience were able to navigate through the decision aid with greater ease and less confusion than first-round users.

### Study design

To pilot test the efficacy of the decision aid, we then conducted a before-after uncontrolled trial at the UNC general internal medicine practice. The practice consists of 75 medical residents, 20 attending physicians, and approximately 12,000 patients, of whom approximately 5,000 are between 50 and 75 years of age.

### Population

We enrolled a convenience sample of patients from June 2003-April 2004. The participants were adults 50 to 75 years old who were cared for in the general internal medicine practice and presented to their provider for a scheduled appointment. Eligibility criteria were: 1) the absence of a personal or family history of colon cancer in a first degree relative; 2) sufficient general health to undergo screening as determined by the research assistant (RA) or primary care provider; and 3) the ability to communicate in English. The RA sought permission from providers and then approached eligible patients and asked them to enroll. Clinic providers also referred patients for participation. If the patient agreed to participate, the RA obtained written informed consent. Both patients who were up-to-date with screening and those who were due for screening were allowed to enroll with the rationale that individuals in both groups could learn more about screening options for their next decision about screening. We defined up-to-date status as having an FOBT in the past year, sigmoidoscopy or barium enema in the past 5 years, or colonoscopy in the past 10 years. If we found in patients' medical records or baseline surveys that they were up-to-date with screening, they were asked to respond as if they were deciding about their next screening opportunity.

### Intervention

Participants viewed the decision aid on a computer in a private area in the clinic either before or after their scheduled appointment. The RA was present during their viewing session. The patients were encouraged to navigate independently through the decision aid and questionnaires, but were offered assistance from the RA if needed.

### Data collection

Patients completed web-based, self-administered questionnaires before and after viewing the decision aid (see Additional file 1). We based the questionnaires' content and format on the questions used in our videotape decision aid trial. After viewing the decision aid, participants indicated their stage of readiness to be screened by choosing one of three color-coded stages: green indicated that they were ready to be screened, yellow that they needed more information, and red that they did not want screening. They completed an additional paper-based questionnaire based on their stage of readiness (Appendix A). We asked patients who were ready to be screened what criteria were most important in deciding on screening and which test they would prefer to have. The RA conducted an electronic chart review 6 months afterwards to determine if CRC screening tests were ordered and completed.

### Outcome measures

The primary outcome measures were: 1) intent to ask providers about screening; and 2) interest in CRC screening. Intent to ask providers for screening was measured on a 4-point Likert scale (1 = not at all likely to ask, 4 = very likely to ask). Patients' interest in being screened for CRC in the next 6 months was also measured on a 4-point Likert scale (1 = not at all interested, 4 = definitely interested).

Secondary outcome measures included: subjective change in knowledge about screening, helpfulness of the information in making a decision about screening, and preferences for shared decision making with their provider.

Other outcome measures were the proportions of CRC screening tests ordered and completed after 6 months. Test ordering was defined as an FOBT order recorded in the clinic's database or a colonoscopy, sigmoidoscopy, or barium enema order in the electronic medical record. Test completion was defined as a completed FOBT recorded in the clinic database or a completed colonoscopy, sigmoidoscopy, or barium enema report in the electronic medical record. Patients choosing a test were then asked to give their main reason for their particular choice.

### Statistical analysis

We examined the characteristics of the sample by using univariate analyses to determine the distribution of each variable. The mean, range, and standard deviation were calculated for continuous variables and frequencies and percentages were tabulated for categorical variables. To assess change in interest and intent to be screened, we used paired t-tests to compare the difference in Likert scores before and after viewing the decision aid. Frequencies and percentages were tabulated for categorical variables in the questionnaires and for test ordering and completion.

Pearson's chi-square test was used to compare the percentage ordering and completing tests by stage of readiness to be screened. We conducted another analysis after excluding patients who were already up-to-date with screening because these patients might be less likely to have tests ordered or completed after viewing the decision aid. We used Pearson's chi-square test to compare the proportion completing tests among patients who had previously been screened, were up-to-date with screening, or had previous conversations about screening with their provider vs. those who had not.

Two-sided p-values < 0.05 were considered statistically significant. Stata version 8.2 (College Station, TX) was used for all analyses. Prior approval for the study was obtained from the University of North Carolina-Chapel Hill Institutional Review Board, and the research was carried out in compliance with the Helsinki Declaration [13].

## Results

### Patient characteristics

Approximately 260 patients were approached and 80 agreed to participate (31% response rate). The main reason for non-participation was lack of time to complete the study during the index visit. The demographic characteristics of participants are shown in Table 1; demographic characteristics were similar for those who were up-to-date with screening and those who were not. The mean age was 60 years. Fifty-nine percent were male and 69% were white. Of the 81% with health insurance, 18% had Medicare and 46% had private insurance. Approximately half of the participants had ever been screened for colon cancer and 18% were up-to-date with screening. Almost two-thirds of patients said that a provider had discussed CRC screening with them in the past.

**Table 1:** Characteristics of the sample (n = 80).

**Characteristic**	**Mean (range) or percent**
Mean age	60 (49–75)
% Male	59
% White	69
% African American	29
% Insured	81
% More than high school education	65
% Self-rated excellent-good health	67
% Screened for colorectal cancer in the past	48
% Up to date with screening	18

### Change in interest in screening and intent to be screened

**Figure 4:**
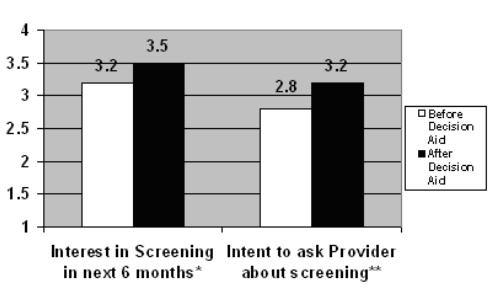
**Change in intent to be screened after viewing the decision aid**. * p = 0.01, paired t-test. Based on 4-point Likert scale, 1 = not at all interested, 4 = very interested ** p < 0.0001, paired t-test. Based on 4-point Likert scale, 1 = not at all likely to ask, 4 = very likely to ask

Interest in screening and intent to ask providers about screening rose significantly after viewing the decision aid. Patients' interest in being screened in the 6 months after viewing the decision aid increased from a mean score of 3.2 before viewing the decision aid to 3.5 afterwards (difference, 0.3; p = 0.01, paired t-test, Figure 4). Patients' intent to ask for screening increased from 2.8 to 3.2 (difference, 0.4; p < 0.0001, paired t-test).

### Process outcomes

Eighty-nine percent said that the information increased their knowledge about colon cancer, 78% said that the information helped them decide whether to be screened, and 90% felt that the amount of information presented was just right. Even among those who were not ready to be screened, most found the aid helpful and reported that it increased their knowledge about colorectal cancer. Ninety percent preferred to make decisions about their health together with their physician. The mean amount of time spent viewing the decision aid was 19 minutes.

### Test ordering and completion

Forty-eight percent had either an FOBT or endoscopy (colonoscopy or sigmoidoscopy) ordered within 6 months of viewing the decision aid (Table 2). Thirty-three percent had an FOBT ordered and 26% had an order for endoscopy. In terms of test completion, 43% of participants completed either FOBT or endoscopy. Twenty-three percent completed FOBT and 26% completed endoscopies, and 2.5% completed both tests. No barium enemas were ordered or performed.

**Table 2:** Six-month follow-up: screening test ordering and completion

	**Total**	**FOBT**	**Colonoscopy/sigmoidoscopy**
**% Ordered**	48	33	26
**% Completed**	43	23	26

All percentages based on n = 80

### Self-reported stage of change and relationship with test completion

When asked about stage of readiness to be screened, 60% chose green (ready to be tested), 18% chose yellow (needed more information) and 22% chose red (not ready to be screened). There was a greater percentage of test ordering and completion among patients choosing green (51% and 43%, respectively) compared to those choosing yellow or red, but these differences did not reach statistical significance (Table 3).

**Table 3:** Test ordering and completion by stage of readiness to be screened

**Stage of Readiness**	**n**	% with test ordered*	% completing test**
**Green: **ready to be tested	47	51	43
**Yellow: **need more Information	18	47	33
**Red: **not ready for screening	15	39	33

*p = 0.59, Pearson's chi-square test

**p = 0.71, Pearson's chi-square test

After excluding patients who were up-to-date with screening, there was no significant change in the percentage of patients having tests ordered and completed by stage of readiness to be screened. Screening test completion rates did not differ significantly between those who were already up-to-date with screening and those who were not in compliance. Individuals who were up-to-date had lower baseline intent and interest scores and fewer were ready to be screened compared to those who were not up-to-date (Table 4). More FOBTs were ordered in those who were up-to-date, but the percentage that had a lower endoscopy was similar to the percentage that had endoscopies among those due for screening. Having a previous history of screening or having previous conversations about screening with a provider did not result in higher test completion rates.

**Table 4:** Intent* and interest** in screening, test ordering and completion, and readiness to be screened by up-to-date^§ ^status

	**Up-to-date (n = 14)**	**Not up-to-date (n = 66)**
**Intent to ask for screening**		
**before viewing aid**	2.4	2.9
**after viewing aid**	2.6	3.3
**Interest in screening**		
**before viewing aid**	2.6	3.3
**after viewing aid**	3.0	3.6
**Readiness to be screened (%)**		
**Ready**	29	66
**Not ready**	43	18
**Need more information**	29	15
**% with lower endoscopy ordered**	21	28
**% with lower endoscopy completed**	21	27
**% with FOBT ordered**	57	21
**% with FOBT completed**	29	17

*Intent to ask for screening was based on the question: "How likely are you, at this visit, to ask your doctor about being tested for colon cancer?" and used a 4-point Likert scale, 1 = not at all likely to ask, 4 = very likely to ask

**Interest in screening was based on the question:"How interested are you in having a test for colon cancer in the next 6 months?" and used a 4-point Likert scale, 1 = not at all interested, 4 = definitely interested

^§^up-to-date: FOBT in the past year, sigmoidoscopy or barium enema in the past 5 years, or colonoscopy in the past 10 years

### Test preferences

We asked patients who were ready to be screened which screening modality they preferred to have. Forty-two percent chose colonoscopy, 20% chose FOBT alone, and 18% chose FOBT in combination with sigmoidoscopy. However, only 28% actually had the test they preferred ordered by their provider. We also asked these patients to choose the most important factor in deciding on a screening test (Appendix A). More than half (54.5%) said that the ability of tests to find cancers or polyps, or test accuracy, was the most important criteria in selecting a screening method.

## Discussion

We developed a computer-based colon cancer screening decision aid and found that it could increase patient interest in screening and intent to be screened. Most participants were ready to be screened after viewing the decision aid, 48% had tests ordered and 43% completed screening tests. These results are similar in magnitude to those from our videotape decision aid trial in which patients' intent to ask providers for screening increased significantly after viewing the aid and 37% completed tests.

In our current study, the computer-based decision aid subjectively improved patients' knowledge about screening and was useful to most in making decisions about screening. Other studies have found that similar tools increased patients' level of knowledge about screening, but effects on screening rates have varied. Zapka et al. conducted a randomized controlled trial of a video on sigmoidoscopy that was mailed to patients in advance of a scheduled visit [14]. A decision aid developed by Meade et al. improved patient knowledge about CRC screening as determined by a change in score from expert-validated pre- and post-tests [15]. Dolan et al. found that patients subjectively reported improved knowledge about CRC screening after using a decision aid [16]. In these studies, there was no difference in screening test ordering and completion between those who viewed the decision aid compared to those who did not [16]. In contrast to these studies that looked at the effect of a decision aid alone, our previous study using a combined intervention of a videotape decision aid and chart marker was able to increase screening test ordering and completion compared to controls [10].

Our computer-based aid differs from other decision aids for CRC screening in that patients were able to interact with the aid via its modular format and choose to view information based on their knowledge needs. Patients in previous trials of decision aids on CRC screening all received the same educational content regardless of their knowledge about screening [10,14-16]. Our computer-based aid was not truly tailored in that the decision aid was not customized to fit individual patients' characteristics. Each patient, however, was able to select the amount and content of information they received. In this way, the information on CRC screening may have achieved greater relevance to patients.

Only 28% of those who were ready to be screened had the test they preferred ordered by their provider. There are a number of possible reasons for the lack of congruence between patient preferences and test ordering. First, some patients may have viewed the decision aid after seeing their provider and thus did not have an opportunity to discuss screening at that visit or another visit within the 6-month follow-up window. Another possibility is that providers may not have been aware of patients' preferences and had not been trained to provide stage-appropriate responses. Third, patients who were already up-to-date with screening may not have had tests ordered. Excluding patients who were up-to-date from the analysis, however, did not increase the proportion of tests ordered, so this explanation is unlikely to account for the low test ordering rates.

Given these results, a patient-oriented decision aid alone may be insufficient to ensure test ordering based on patient preferences or increase test ordering and completion to desired levels; multifaceted interventions that target a combination of providers, patients, or office systems may be more likely to increase screening rates [17]. Interventions such as physician prompts and standing orders can increase performance of preventive care, including cancer screening [18,19]. Standing orders are another potential component of a multifaceted intervention. In a standing orders protocol, a nurse initiates test ordering based on patient preferences and a practice-approved protocol. Implementing the decision aid with office-system interventions may help improve rates of screening test ordering.

The proportion of tests ordered and completed for patients who answered green was higher than for patients who answered yellow or red, but the differences were not statistically significant. The study with its small sample size may have lacked power to detect a significant difference between the groups. In addition, approximately 40% of patients choosing red had tests ordered and one-third completed screening tests. In our previous videotape decision aid study, only 7% of patients choosing red had tests ordered and 4% completed tests. The seeming disconnect between patient interest and provider ordering in the current study is concerning and may be due to poor patient-provider communication about preferences or patients who changed their mind about screening after completing the questionnaire. More research needs to be done to determine why patients who were uncertain or not ready for screening had tests ordered and completed.

Of the patients who were ready for screening, most rated the ability to find cancer, or accuracy, as the most important factor in deciding on a test. Ling et al. previously found that most patients rate accuracy as the most important feature of a CRC screening test, but that providers thought that discomfort in undergoing a test was most important to patients [20]. Providers should be aware that many individuals value the accuracy of screening methods and counsel their patients accordingly.

There are a number of limitations to this study. Foremost, it was an uncontrolled trial without a comparison group, so it is unclear whether the proportion of patients having tests ordered and completed over 6 months represents an increase compared to the usual care of patients who did not view the decision aid but were otherwise eligible for the study. Our results, however, are fairly comparable to those from our videotape decision aid randomized trial conducted in three central North Carolina private primary care practices: overall there was a net 0.6 unit increase in intent to be screened after the decision aid. Among patients viewing the videotape decision aid, 47% of individuals had screening ordered compared to 26% of controls, and 37% completed tests vs. 23% of controls. We chose to use an uncontrolled design as the first phase of testing to evaluate whether the aid could increase interest in screening and was useful to patients in choosing a screening modality. Whether patients' increased interest in screening after viewing the computer-based decision aid can lead to an improvement in screening rates cannot be determined from this pilot study; this question will be better addressed in a larger, multi-center randomized trial with screening test completion as the main outcome.

Other limitations were the use of a convenience sample and selection bias. Given the volunteer study population with some subjects referred by physicians and the low response rate, the responses of those who chose to participate may be different from those who did not participate. Our results also could have been affected by the fact that almost half of the participants were previously screened and 18% were up-to-date with screening. We performed additional analyses excluding those who were up-to-date and did not find a change in the percentage of tests ordered and completed. There were also some differences in outcomes between those who were up-to-date and those who were not. Individuals who were up-to-date had lower mean intent and interest scores at baseline and after watching the aid, and less were ready to be screened after watching the aid compared to those who were not up-to-date (Table 4). These results are based on small numbers, however, and should be interpreted with caution.

Although patients were able to choose different videos in the decision aid, we did not track which segments were viewed by patients. Tracking may have provided additional information on how individual use of the decision aid was related to change in interest, test preferences, or test completion. However, mean viewing time was 19 minutes, indirectly suggesting that patients were accessing a significant portion of the content.

This study did not objectively measure screening knowledge before and after the aid, its effect on decisional conflict, or changes in anxiety or satisfaction with decisions, other important measures of a decision aid's effectiveness [8]. In this pilot study conducted in a busy primary care practice, we chose to focus on whether the aid could increase interest in screening and was useful in deciding on a screening modality. Future studies should assess whether this decision aid can decrease decisional conflict and improve objective knowledge about screening.

Because our study was conducted at a single site, our study findings may not be generalizable to other populations. Those in our convenience sample had high levels of education, most had insurance, and many had prior experience with screening. Other patient populations, including individuals not currently receiving regular medical care, might respond differently to the decision aid.

Although Medicare, Medicaid, and most private insurers cover CRC screening [21], cost may be an important issue for patients. We did not collect information on which tests were covered by patients' insurance carriers, co-pays and deductibles, or the importance of cost in patients' decisions about screening. Evaluating the effect of different levels of co-payment on patient preferences is an important area for future research.

A final limitation is that the decision aid may be somewhat challenging for those with limited computer skills. Although we did not objectively measure how many patients needed assistance, we observed that most patients completed the aid independently and required limited, if any, computer assistance. Whether the additional benefits of the web-based format outweigh the greater requirements for computer skill requires further research. We have developed a DVD version of the decision aid that preserves the ability to self-navigate but may be easier for computer-inexperienced users or those without access to a computer.

## Conclusion

This computer-based decision aid on colorectal cancer screening increased patient interest in screening and intent to be screened. Most patients could independently navigate through the menu of choices to select segments that met their knowledge needs. It is interactive and takes approximately 20 minutes of patient time.

There are many ways in which the decision aid can be incorporated into primary care practice. Because the decision aid explains the importance of CRC screening and describes each test with its risks and benefits, it can potentially save providers time in counseling patients about screening. Internet- and DVD versions of the decision aid are currently in production, and we plan to produce a Spanish language version that will allow for dissemination of the aid to Spanish-speaking patients. The decision aid could be watched by patients at home in preparation for a provider appointment, a process we are currently testing in another study. In the office, nurses can identify patients at triage who are due for screening and direct them to watch the decision aid prior to an upcoming visit or while waiting to see their provider.

In this pilot study, a computer-based, patient-directed decision aid increased patient interest in colorectal cancer screening and subjectively improved knowledge about screening options. Most patients were ready to be screened after viewing the decision aid but only half the patients who wanted to be screened had tests ordered. Future research needs to be done to determine whether implementation of the decision aid with other interventions can effectively raise screening rates in a primary care setting.

## Competing interests

The author(s) declare that they have no competing interests.

## Authors' contributions

JK participated in data collection, performed the statistical analysis, and drafted the manuscript. AW and SH participated in data collection. CL helped conceive of the study, participated in the design of the study and participated in the drafting and editing of the decision aid. MC, LS, and BF participated in the drafting and editing of the decision aid. SG programmed the decision aid interface. RM participated in the design of the decision aid. MP conceived of the study, participated in the drafting and editing of the decision aid, designed and coordinated the study, and helped to draft the manuscript. All authors read and approved the final manuscript.

## Pre-publication history

The pre-publication history for this paper can be accessed here:



## Supplementary Material

Additional File 1 Decision aid questionnaires Click here for file
