# Supplementary material for: Development and initial testing of a computer-based patient decision aid to promote colorectal cancer screening for primary care practice
Source: BMC Med Inform Decis Mak. 2005 Nov 28;5:36. doi: 10.1186/1472-6947-5-36 (PMC1318488; doi:10.1186/1472-6947-5-36)
Supplement: Additional File 1 — Decision aid questionnaires [file 1472-6947-5-36-S1.doc]

### Questionnaire 1: Administered prior to viewing decision aid

**Question 1. In general, how would you rate your health?**

1. Excellent
2. Very Good
3. Good
4. Fair
5. Poor

Question 2. Has a doctor or nurse ever talked to you about having a test for colon cancer?

1. Yes
2. No
3. I am not sure

**Question 3. Have you ever had a test for colon cancer?**

1. Yes
2. No
3. I am not sure

**Question 4. Have you had a stool test for blood? (You take this test home and put a small sample of your stool on it and either mail or return it to your doctor.)**

1. Yes, more than a year ago
2. Yes, within the last year
3. No, I have never had this test
4. I am not sure

**Question 5. Have you had a flexible sigmoidoscopy? (This test requires a tube with a light being inserted into your rectum. This allows the doctor to see the lower third of your colon. It takes about 15 minutes. You are not given medicine to make you sleepy with this test.)**

1. Yes, more than five years ago
2. Yes, within the last five years
3. Yes, but I can’t remember when
4. No, I have never had this test
5. I am not sure

**Question 6. Have you had a barium enema test for colon cancer? (This test requires an x-ray taken after a special enema is given.)**

1. Yes, more than five years ago
2. Yes, within the last five years
3. Yes, but I can’t remember when
4. No, I have never had this test
5. I am not sure

**Question 7. Have you had a colonoscopy? (This test requires a tube with a light that is inserted into your rectum. It allows the doctor to see your entire colon. You are given medicine to make you sleepy with this test.)**

1. Yes, more than ten years ago
2. Yes, within the last ten years
3. Yes, but I can’t remember when
4. No, I have never had this test
5. I am not sure

**Question 8. How interested are you in having a test for colon cancer in the next 6 months?**

1. Very interested
2. Somewhat interested
3. Not very interested
4. Not at all interested

**Question 9. How likely are you, at this visit, to ask your doctor about being tested for colon cancer?**

1. Very likely
2. Somewhat likely
3. Not very likely
4. Not at all likely

**Question 10. When I need to make a decision about my health**

1. I prefer to make the decision myself.
2. I prefer to make the decision after listening to my doctor’s opinion.
3. I prefer that my doctor and I make the decision together.
4. I prefer that my doctor make the decision after considering my opinion.
5. I prefer that my doctor make the decision.

**Question 11. Are you**

1. White
2. Black/African American
3. Hispanic
4. Asian/Pacific Islander
5. Other

**Question 12. What is the highest grade of school you have completed?**

1. Less than 9^th^ grade
2. Some high school but did not graduate
3. 12^th^ grade (graduated high school) or GED
4. Some college or technical training
5. 4 year college degree or more

**Question 13. Do you have health insurance?**

1. Yes
2. No

**Question 14. Which of the following health insurance do you have? (please check all that apply)**

1. Medicare
2. Medicaid
3. Private Insurance, for example, Blue Cross/Blue Shield
4. I am not sure

## Questionnaire 2: Administered after viewing the decision aid

**Question 1. Right now, how interested are you in having a test for colon cancer in the next 6 months?**

1. Definitely interested
2. Somewhat interested
3. Undecided
4. Not very interested
5. Not at all interested

**Question 2. How likely are you now to ask your doctor about a test for colon cancer on this visit?**

1. Very likely
2. Somewhat likely
3. Not very likely
4. Not at all likely

**Question 3. The amount of information I received was:**

1. Too much
2. Too little
3. About right

**Question 4. The information presented increased my knowledge about colon cancer.**

1. Strongly agree
2. Agree
3. Neither agree nor disagree
4. Disagree
5. Disagree strongly

**Question 5. The information presented helped me decide whether to be screened or not.**

1. Strongly agree
2. Agree
3. Neither agree nor disagree
4. Disagree
5. Disagree strongly

**Question 6. Rate yourself:**

1. Green: I am ready to be tested
2. Yellow: I think I would like to be tested, but I need more information
3. Red: I don’t want to be tested now

Questionnaires 3, 4, 5: Green (#1G), Yellow (#1Y), Red (#1R) administered based on patient’s self rated readiness to be screened

For Office Use Only

Patient ID ____________________

Practice ID ___________________

RA# ________________________

Date Collected: ____/____/____

mm dd yy

##### CHOICE

###### Patient Questionnaire #1G

# *Now that you've seen the CHOICE decision aid, we just have a few final questions.*

**Please circle one response for the following questions.**

1. **How important do you think it is to your doctor that you have a regular test for colon cancer?**

a. very important

b. somewhat important

c. not very important

d. not important at all

e. don't know

1. **Did you and your doctor discuss testing for colon cancer on your visit today?**

a. yes, a good discussion

b. yes, but only a brief mention

1. no (skip to question 4)
2. have not seen doctor (skip to question 5)
3. **If you had a discussion about colon cancer screening, who brought it up?**
   1. I brought it up.
   2. My doctor brought it up.
4. **On today's visit, did you and your doctor decide that you would have a colon cancer screening test ordered?**
5. yes
6. no
7. **The length of the video clips were:**
8. too long
   1. too short
   2. just right

##### CHOICE

For Office Use Only

Patient ID ____________________

Practice ID ___________________

RA# ________________________

Date Collected: ____/____/____

mm dd yy

###### Patient Questionnaire #1G

continued

1. **The information presented helped me decide which test to have.**
   1. strongly agree
   2. agree
   3. neither agree nor disagree
   4. disagree
   5. disagree strongly
2. **What was the most important factor for you in deciding which test to have?**
3. the preparation for the test
4. whether or not I receive medication during the test to make me sleepy
5. possible discomfort during the test
6. amount of time required
7. ability of the test to find cancers or polyps at one point in time
8. possible complications from the test
9. **Which test would you like to have?**
10. stool test for blood (FOBT) every year alone
11. flexible sigmoidoscopy every 5 years alone
12. stool test for blood (FOBT) every year and flexible sigmoidoscopy every 5 years
13. colonoscopy every 10 years alone
14. barium enema every 5 years alone
15. I can’t decide which test to have

**Thank you for your participation!**

##### CHOICE

For Office Use Only

Patient ID ____________________

Practice ID ___________________

RA# ________________________

Date Collected: ____/____/____

mm dd yy

###### Patient Questionnaire #1Y

# *Now that you've seen the CHOICE decision aid, we just have a few final questions.*

**Please circle one response for the following questions.**

**1. How important do you think it is to your doctor that you have a regular test for colon cancer?**

a. very important

b. somewhat important

c. not very important

d. not important at all

e. don't know

1. **Did you and your doctor discuss testing for colon cancer on your visit today?**
   1. yes, a good discussion
   2. yes, but only a brief mention
   3. no (skip to question 4)
   4. have not seen doctor (skip to question 5)
2. **If you had a discussion about colon cancer screening, who brought it up?**
   1. I brought it up.
   2. My doctor brought it up.
3. **On today's visit, did you and your doctor decide that you would have a colon cancer screening test ordered?**
4. yes
5. no
6. **The length of the video clips were:**
   1. too long
   2. too short
   3. just right

##### CHOICE

For Office Use Only

Patient ID ____________________

Practice ID ___________________

RA# ________________________

Date Collected: ____/____/____

mm dd yy

Patient Questionnaire #1Y

continued

1. **Which of the following things is most important to you in deciding about whether or not to be tested for colon cancer?**
   1. not sure if colon cancer is an important issue for me or not
   2. not sure if getting screened will help me or not
   3. concerned about the test preparation
   4. concerned about discomfort from the tests
   5. concerned about time missed from work
   6. concerned about the costs I will have to pay
   7. concerned about possible complications from being tested
   8. can’t decide which test to have
   9. I don’t have enough information to decide about being tested
   10. other: __________________________________________________________

**Thank you for your participation!**

##### CHOICE

For Office Use Only

Patient ID ____________________

Practice ID ___________________

RA# ________________________

Date Collected: ____/____/____

mm dd yy

###### Patient Questionnaire #1R

# *Now that you've seen the CHOICE decision aid, we just have a few final questions.*

**Please circle one response for the following questions.**

**1. How important do you think it is to your doctor that you have a regular test for colon cancer?**

a. very important

b. somewhat important

c. not very important

d. not important at all

e. don't know

**2.** **Did you and your doctor discuss testing for colon cancer on your visit today?**

- 1. yes, a good discussion
  2. yes, but only a brief mention
  3. no (skip to question 4)
  4. have not seen doctor (skip to question 5)

**3. If you had a discussion about colon cancer screening, who brought it up?**

- 1. I brought it up.
  2. My doctor brought it up.

1. **On today's visit, did you and your doctor decide that you would have a colon cancer screening test ordered?**
   1. yes
   2. no
2. **The length of the video clips were:**
   1. too long
   2. too short
   3. just right

##### CHOICE

For Office Use Only

Patient ID ____________________

Practice ID ___________________

RA# ________________________

Date Collected: ____/____/____

mm dd yy

Patient Questionnaire #1R

continued

1. **Which of the following things best describes why you are not interested in being screened for colon cancer at this time?**
   1. colon cancer is not an important issue for me
   2. I am not at risk for colon cancer
   3. concerned about the test preparation
   4. the test preparation makes me not want to be screened
   5. the discomfort from the test makes me not want to be screened
   6. missing time from work makes me not want to be screened
   7. the costs I have to pay makes not want to be screened
   8. I want to be screened, but don’t want to schedule a test today
   9. possible complications from being tested make me not want to be screened
   10. my doctor hasn’t said that I need to be screened
   11. I need more information before I decide about screening
   12. other: ____________________________________________________

**Thank you for your participation!**
